# Effect of MU‐weighted multi‐leaf collimator position error on dose distribution of SBRT radiotherapy in peripheral non‐small cell lung cancer

**DOI:** 10.1002/acm2.13061

**Published:** 2020-10-31

**Authors:** AiHui Feng, Hua Chen, Hao Wang, HengLe Gu, Yan Shao, YanHua Duan, YanChen Ying, Ning Jeff Yue, ZhiYong Xu

**Affiliations:** ^1^ Department of Radiation Oncology Shanghai Chest Hospital Shanghai Jiao Tong University Shanghai China; ^2^ Shcool of Physics and Technology University of Wuhan Wuhan China; ^3^ Department of Radiation Oncology Rutgers Cancer Institute of New Jersey Rutgers University New Brunswick NJ USA

**Keywords:** auto‐planning, MLC position error, MU‐weighted, SBRT

## Abstract

**Purpose:**

Position accuracy of the multi‐leaf collimator (MLC) is essential in stereotactic body radiotherapy (SBRT). This study is aimed to investigate the dosimetric impacts of the MU‐weighted MLC positioning uncertainties of SBRT for patients with early stage peripheral non‐small cell lung cancer (NSCLC).

**Methods:**

Three types of MLC position error were simulated: Type 1, random error; Type 2, system shift, in which both MLC banks shifted to the left or right direction; and Type 3, in which both MLC banks moved with same magnitudes in the opposite directions. Two baseline plans were generated: an automatic plan (AP) and a manually optimized plan (MP). Multi‐leaf collimator position errors were introduced to generate simulated plans with the preset MLC leaf position errors, which were then reimported into the Pinnacle system to generate simulated plans, respectively. The dosimetric parameters (CI, nCI, GI, etc.) and gEUD values of PTV and OARs were calculated. Linear regression between MU‐weighted/unweighted MLC position error and gEUD was performed to obtain dose sensitivity.

**Results:**

The dose sensitivities of the PTVs were −4.93, −38.94, −41.70, −55.55, and 30.33 Gy/mm for random, left shift, right shift, system close, and system open MLC errors, respectively. There were significant differences between the MU‐weighted and the unweighted dose sensitivity, which was −38.94 Gy/mm vs −3.42 Gy/mm (left shift), −41.70 Gy/mm vs −3.56 Gy/mm (right shift), −55.55 Gy/mm vs −4.84 Gy/mm (system close), and 30.33 vs 2.64 Gy/mm (system open). For the system open/close MLC errors, as the PTV volume became larger, the dose sensitivity decreased. APs provided smaller dose sensitivity for the system shift and system close MLC errors compared to the conventional MPs.

**Conclusions:**

There was significant difference in dose sensitivity between MU‐weighted and unweighted MLC position error of SBRT radiotherapy in peripheral NSCLC. MU is suggested to be included in the dosimetric evaluation of the MLC misalignments, since it is much closer to clinical radiotherapy.

## 1 INTRODUCTION

Lung cancer is one of the major malignant tumors with high morbidity and mortality in China and worldwide. The incidence rate of lung cancer is steadily increasing.^1^ With the advancement of radiotherapy, stereotactic body radiotherapy (SBRT) has become an increasingly common treatment option for patients with non‐small cell lung cancer (NSCLC), with comparable clinical outcomes to surgery.^2^, ^3^, ^4^ For patients with inoperable NSCLC, SBRT is often the critical alternative therapy.

Stereotactic body radiotherapy demands much more stringent dose constraints to both the target volumes and critical normal tissues, which in turn requires a high‐quality treatment plan, and efficient treatment planning process and technologies. In order to improve quality, efficiency, and consistency in treatment planning, automated treatment planning systems (ATPs) have gained wide interest in the radiation oncology and medical physics communities.^5^, ^6^, ^7^ The automatic planning systems can in principle achieve highly consistent treatment automatic plans (APs) in which the target coverage can be significantly improved at the little expense of planning time compared to manual plans (MPs).^8^ However, APs automatically generate many artificial dose limiting structures and corresponding dose parameters,^9^, ^10^, ^11^ which might increase the complexity of the plan.

Treatment options for SBRT include three‐dimensional conformal radiation therapy (3D‐CRT), step‐and‐shoot intensity‐modulated radiation therapy (IMRT), and volumetric‐modulated arc therapy (VMAT). In our center, step‐and‐shoot IMRT is routinely used for SBRT treatments. In the treatment delivery of IMRT, the multi‐leaf collimator (MLC) leaves are divided into many subfields, and the position accuracy of the MLC leaves can directly cause dose deviations from the desired for both the target volumes and organs at risk (OARs). By analyzing the MLC log files of IMRT delivery treatments, Olasoloalonso et al.^12^ found that the root mean square error (RMSE) of MLC position values were 0.306 mm for Clinac linacs (Varian Medical Systems, CA, USA) in IMRT treatments and 0.038 mm for TrueBeam linacs (Varian Medical Systems, CA, USA) in VMAT treatments. This study was based on prior work on MLC error during IMRT delivery. Oliver et al.^13^ reported that for systematic MLC gap open errors, the dose sensitivity was 8.2% per mm and for MLC gap close errors the dose sensitivity was −7.2% per mm.

We found that MU, one of the complexity levels, has an impact on dose distribution. For example, if the MU of a single beam is large, though the MLC error of this beam is small, it still has quite a large effect on dose distribution. Furthermore, the dosimetric impact of MLC positional errors is important when it comes to SBRT since the delivery requirements can be more stringent. In addition, the previous studies were all conducted on treatment deliveries designed with manual plans and did not involve fast‐growing automatic plans. Hence, there is a need to investigate the dosimetric impacts of MU‐weighted MLC position errors not only on the MPs but also on APs for SBRT treatments.

The aim of this study is to explore the effects of MU‐weighted MLC position error on dose distributions of SBRT in APs for NSCLC, and compare with MPs. We explored the differences in dose sensitivity between APs and MPs for three types of MLC errors, and tried to find out whether MU‐weighted is necessary for dosimetric evaluation of the MLC misalignments.

## 2 MATERIALS AND METHODS

### 2.A Patient selection and contouring

A total of ten patients were selected for this study. All patients were diagnosed with clinically stage I–IIA peripheral NSCLC and underwent CT simulation scans using a SOMATOM Definition AS (Siemens Healthcare Gmbh, Erlangen, Bayern, Germany). The slice thickness of CT images was 3 mm. Four‐dimensional CT images were acquired to allow delineation of internal target volume (ITV) for lung SBRT. PTV included the entire delineated ITV plus a 5‐mm margin. All the patients received SBRT on a linear accelerator equipped with CBCT using online IGRT (Varian, Palo Alto, CA) for each treatment fraction.

### 2.B Treatment planning of SBRT

Two SBRT IMRT plans, an automatic plan and a manual plan, were generated for each of the ten patients, respectively. The plans were generated using Pinnacle^3TM^ treatment planning system (TPS, v9.10, Philips Medical Systems, Cleveland, USA) for an Edge linear accelerator (Varian, Palo Alto, CA) equipped with 120 MLC leaves (Millennium MLC) and 6 MV photon beam. The autoplanning module of Pinnacle^3TM^ TPS is based on progressive automatic algorithm,^14^, ^15^, ^16^ which is a fully integrated module, similar to the manual inverse optimizer module. During AP module, individual optimization goals, constraints, and weights are automatically added and adjusted. In addition, the optimizer is automatically run multiple times with adjustments being made during and between optimization processes.^8^ APs and MPs were created using the same set of 10 coplanar beams and other basic beam parameters. For each plan, optimizations were performed with a direct machine parameter optimization (DMPO) algorithm and dose distributions were calculated using the collapsed cone convolution algorithm (CCC) with a calculation grid of 2 mm. The prescribed dose for lung SBRT was 50 Gy (10 Gy/fraction) to PTV; the dose limits of OARs are defined according to RTOG 0813 protocol.^17^ Minor deviations were allowed only if it is necessary to achieve the dose constraints for the OARs and if the maximum dose remained within the ITV.

### 2.C Simulation of MLC position error in SBRT plans

**Fig. 1:**
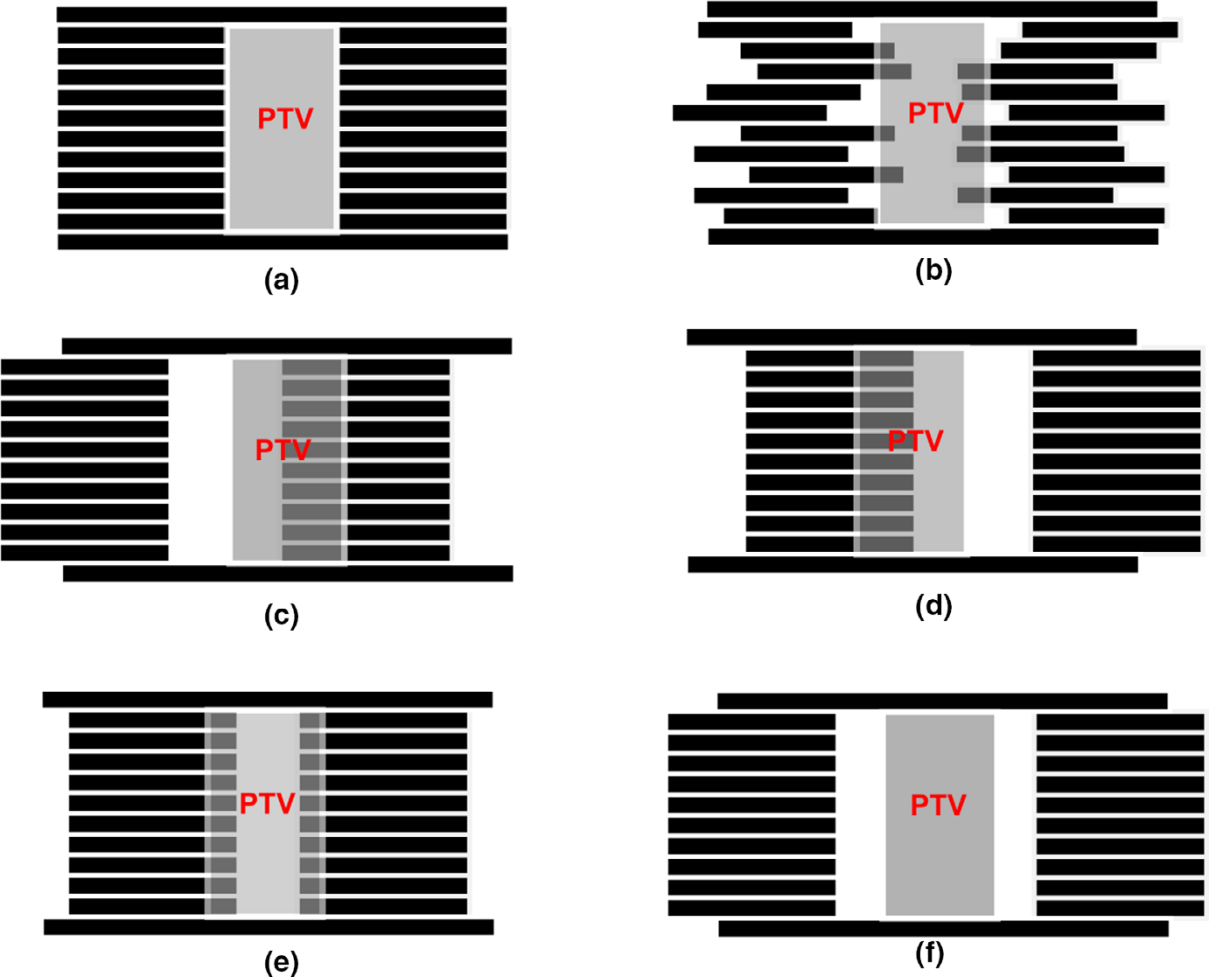
Three types of MLC position errors introduced to each SBRT plans. (a) baseline plan; (b) Type 1, random error; (c) Type 2a, left shift; (d) Type 2b, right shift; (e) Type 3a, system close, and (f) Type 3b, system open.

Three types of MLC position errors were investigated in this study. As an example, Fig. 1(a) shows the leaf positions in a plan. Type 1 errors were random errors that were simulated and introduced by sampling a Gaussian function with error magnitude equal to the standard derivation. In simulated plans, the moving leaf banks were made to randomly deviate from the planned positions, either extending over or retracting back as shown in Fig. 1(b). Type 2 errors were system leaf shifts: all the MLC leaves shifting to the left [Type 2a, shown in Fig. 1(c)] or the right [Type 2b, shown in Fig. 1(d)] by the same error magnitudes, and the subfield area sizes remained unchanged during this error simulation. For Type 3 errors, both MLC leaf banks move with a same magnitude in the opposite directions, resulting in the decrease and increase of the MLC leaf gap, and were analyzed as Type 3a (system close) and Type 3b (system open), respectively, as shown in Figs. 1(e) and 1(f).

Five error magnitudes, namely 1, 2, 3, 4, and 5 mm, were simulated for each type of MLC position error. The baseline plans were first exported out from the TPS then imported into an in‐house software system developed with C++ (Visual C++ 6.0). With the in‐house software system, the beam delivery MLC leaf positions were modified with the simulated errors. Afterward, the simulated plans with the MLC leaf errors (named as plan trials) were imported back into the Pinnacle TPS to generate simulated full plans. If the simulated MLC positions resulted in a negative leaf gap (i.e., leaf collision), the MLC positions were re‐adjusted for the gap to be zero.

For each patient, 50 plan trials with different MLC position errors were generated, including five different error magnitudes for five different scenarios of error in plans generated with the AP and MP methods. A total of 500 simulated plans were created for this study.

### 2.D Dose distribution analysis of the SBRT plans

A total of 50 Gy in five fractions was prescribed to the PTV. The dosimetric parameters selected for evaluation of doses of target include conformity index (CI), new conformity index (nCI), D2cm, gradient index (GI), and generalized equivalent uniform dose (gEUD). The CI was computed as:$\text{CI}=\frac{prescription\;isodose\;volume\left(PIV,\;cm^{3}\right)}{tumor\;volume\;encompassed\;prescription\;isodose\;line\left(TIV,\;cm^{3}\right)}$. The nCI was calculated as: *nCI = CI_coverage_*, in which coverage was defined as the ratio of target volumes covered with prescription dose to the target volume. The D_2cm_ is the maximum dose, in percent of prescription dose, at 2 cm from the PTV in any direction. The definition of GI was the ratio of the volume covered by 50% of the prescription dose to the volume covered by the prescription dose. In this study, the GI was computed as: $GI=\frac{R_{50\%}}{R_{100\%}}$, where R_50%_ is the ratio of 50% prescription isodose volume to the PTV and R_100%_ is the ratio of 100% prescription isodose volume to the PTV, which is mathematically equivalent to the previous definition. The gEUD was calculated as:$gEUD={\left(\frac{1}{m}\sum_{i=1}^{m}d_{i}^{a}\right)}^{\frac{1}{a}}$, where, m is the number of voxels in the anatomical structure of interest, d_i_ is the dose on the ith voxel, and **a** is a tumor or normal tissue specific parameter. According to the research of Chapet et al,^18^ the value of **a** was set as −20 for PTV.

Evaluations of doses to OAR included the maximum dose (D_max_) of spinal cord, doses to the total lung, including the mean lung dose (MLD), and the volumes receiving at least 10 Gy (V_10_) and 20 Gy (V_20_).The gEUD values of total lung and spinal cord were calculated, with the parameter **a** set to 1 and 20, respectively, according to the study of Mihailidis et al.^19^ and Rangel et al.^20^

The percentage change [Eq. (1)] was used to estimate the relative error between a simulated plan and the corresponding baseline plan.

(1) $$\Delta X=Relative\;Percentage\;Change=\frac{X_{\mathrm{Error}}‐X_{\mathrm{Base}}}{X_{\mathrm{Base}}}\times 100\%$$

where X represents a parameter used in the evaluations, X_Error_ is the parameter of a simulation plan, while X_Base_ is the parameter of the corresponding baseline plan.

### 2.E Dose sensitivity study of MLC position error

To investigate the dependence of dosimetric change with the MLC position error, one quantity, dose sensitivity, was introduced. First, ΔgEUD, the absolute difference between the gEUD value of a simulated plan and the corresponding baseline plan was calculated, and the ΔgEUD values and the corresponding magnitudes of MLC errors were fitted using the linear regression method. The slope of the linear regression fitting function for the group was defined as the dose sensitivity to MLC position errors. The unit of the dose sensitivity is Gy/mm.

### 2.F Linear regression study of MU‐weighted MLC position error

For any IMRT delivery, there are always multiple MLC segments for the generation of desired fluence patterns, and each of the segments is dose or MU weighted. It is intuitive that the impacts of the MLC position errors depend on not only the geometric magnitude of the errors of each segment but also on the corresponding dose or MU weighting factor. To a certain extent, the MU‐weighted magnitude of MLC error could be more a dominant factor affecting dose distribution than the MLC position error itself. The impact of the MU weighted magnitude of the MLC position error was investigated, in addition to the MLC position error alone. Two kinds of linear regression were performed. The first one only considered the linear relationship between the MLC position error and the ∆gEUD. The fitting formula was *∆gEUD = k* × *MLCPE + b*. where MLCPE stands for the magnitudes of MLC position errors, and k is the slope of linear fit which namely is the dose sensitivity.

The second fit took MU of a single beam into account and normalized it to the total MU of the patient. The fitting formula was expressed as:

$$\Delta gEUD_{i}=k_{\mathrm{ij}}\times \frac{MU_{\mathrm{ij}}}{\sum_{j=1}^{j=n}MU_{\mathrm{ij}}}\times MLCPE_{\mathrm{ij}}+b_{\mathrm{ij}}$$

where $k_{\mathrm{ij}}$ represents the dose sensitivity of jth beam of ith patient, $MU_{\mathrm{ij}}$ represents the jth subfield MU of the ith patient, n represents the total number of subfields of the ith patient, $\sum_{j=1}^{j=n}MU_{\mathrm{ij}}$ represents the total MU of ith patient,$MLCPE_{\mathrm{ij}}$ represents the position error of the jth subfield of the ith patient, $b_{\mathrm{ij}}$ represent the intercept of jth beam of ith patient.

Secondly, the dose sensitivity of ith patient was calculated as:

$$k_{i}=\frac{1}{n}\sum_{j=1}^{j=n}k_{\mathrm{ij}}.$$

Finally, we calculated the dose sensitivity of each patient and then took the mean value to get the mean dose sensitivity of ten patients.

It should be noted that since it is hard to track the absolute value of random error in our study, we had to use the maximum error (1, 2, 3, 4, 5 mm) as $MLCPE_{\mathrm{ij}}$ instead of true random error.

### 2.G Statistical analysis

Data analysis was performed using SPSS software version 20.0 (SPSS, IBM Corp, Armonk, NY). Wilcoxon’s signed‐rank test was performed for two kinds of linear regressions and different planning methods, and *P *< 0.05 was considered to be statistically significant. The linear regression fits were analyzed by using Software package Origin (version 9.0).

## 3 RESULTS

### 3.A Evaluation of dosimetric parameters

**Fig. 2:**
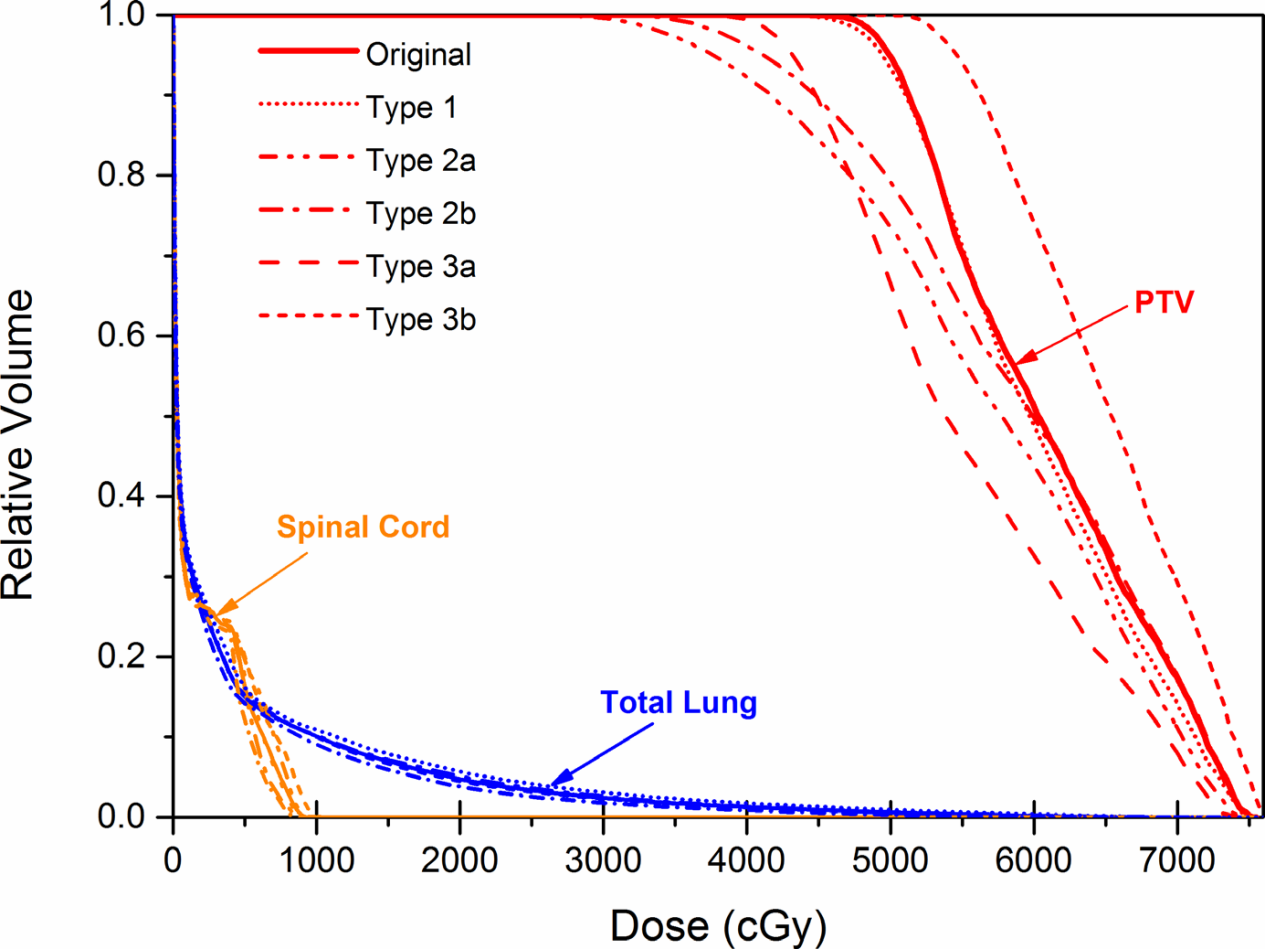
A sample dose–volume histogram of patient 3, baseline AP plan (solid), Type 1: random (short dot), Type 2a: left shift (dash dot dot), Type 2b: right shift (dash dot), Type 3a: systematic close (dash), and Type 3b: systematic open (short dash).

Figure 2 presented the dose–volume histograms (DVH) of PTV, spinal cord, and total lung for the baseline AP plan and the simulated plans with an MLC position error of 5 mm for a typical patient. From the figure, it is evident that the DVH curves of Type 1 MLC error basically coincided with those of the baseline plan. However, the errors of Type 2 and Type 3, which are systematic errors, induced remarkable dosimetric changes. As we can see that two dash‐dot curves of Type 2a and Type 2b MLC errors are not completely coincident, there are dosimetric differences between Type 2a and Type 2b errors.

**Fig. 3:**
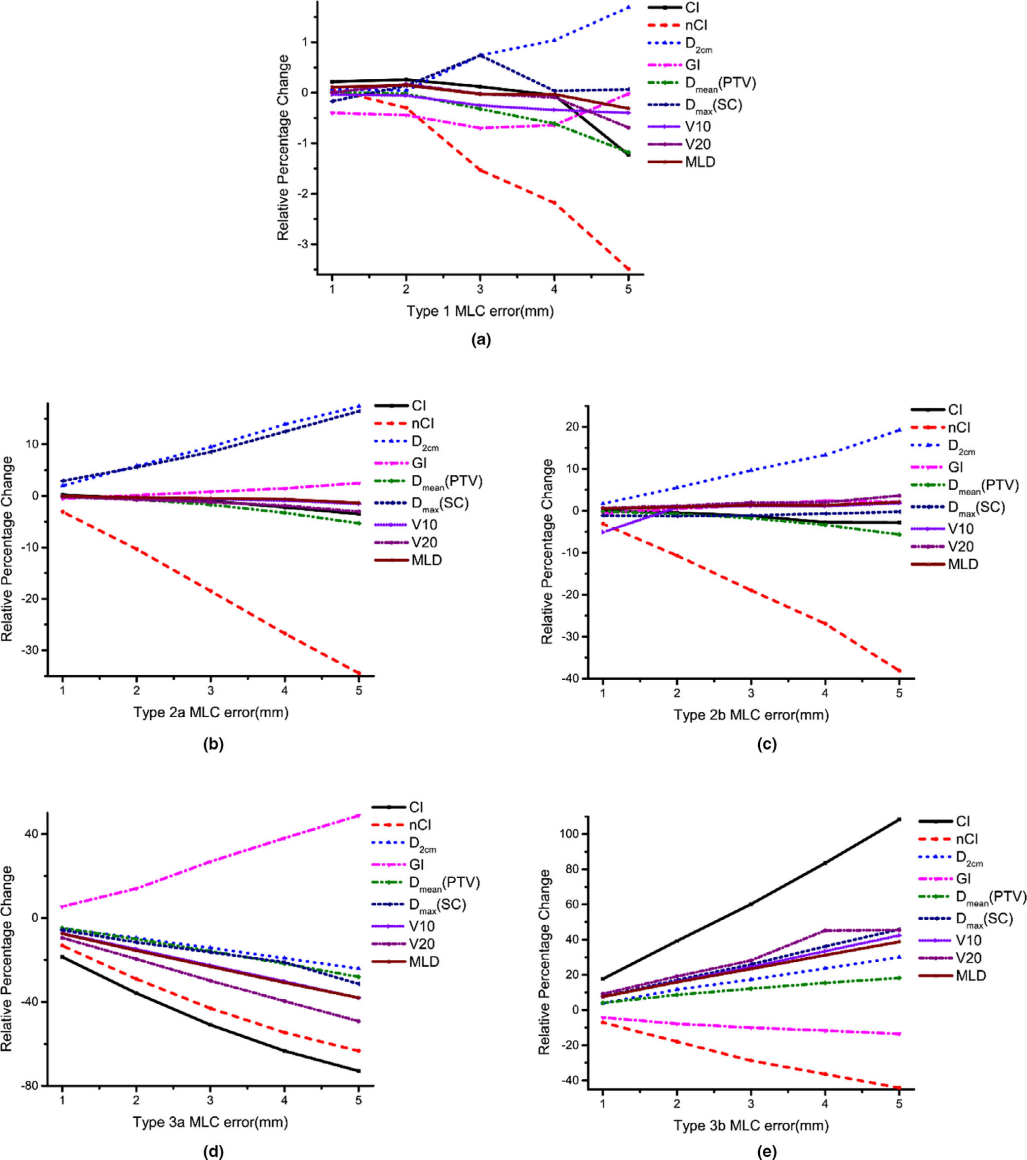
Relative percentage change of CI, nCI, D_2cm_, GI, and D_mean_ of PTV, the D_max_ of the spinal cord, the V_10_, V_20,_ and MLD of the total lung for 3a: random error, 3b: left shift, 3c: right shift, 3d: system close, and 3e: system open.

Figure 3 showed the percentage changes (∆X) of CI, nCI, D_2cm_, GI, and D_mean_ for PTV, the D_max_ of the spinal cord, the V_10_, V_20,_ and MLD of the total lung, respectively.

As is observed in Fig. 3(a), the random error (Type 1) had no obvious effects on the doses of PTV and OARs. The dose parameter with the biggest deviation was nCI. When the simulated Type 1 error magnitude of MLC position was 1, 2, 3, 4, and 5 mm, the absolute change of PTV D_mean_ was 0.012, −0.02, −0.32, −0.61, −1.18%, respectively, showing a near‐linear correlation with MLC misalignments.

For the left shift MLC error (Type 2a), the dosimetric parameter of the largest percent change was nCI, followed by D_2cm_, and the MLD had the minimum change. When both leaves of MLC shifted by 1, 2, 3, 4, and 5 mm in the left direction, the corresponding percentage change of PTV D_mean_ was −0.08, −0.69, −1.70, −3.27, −5.32%, respectively. As the magnitude of the system shift error increased, the deviation of PTV D_mean_ also increased.

Similarly, the largest change of dosimetric parameter for the right shift MLC error (Type 2b) was nCI, followed by D_2cm_. When the leaves of MLC shifted by 1, 2, 3, 4, and 5 mm in the left direction, the corresponding percentage change of PTV D_mean_ was −0.12, −0.74, −1.76, −3.38, and −5.63%, respectively.

When the MLC leaf were shifted to the left direction (Type 2a), the D_max_ of spinal cord increased, the V_10_, the V20, and MLD of total lung decreased. When the leaves moved to the right direction (Type 2b), the changes of OARs were completely opposite. With the introduction of Type 2 error, no matter which direction the MLC leaves shifted to, CI and nCI decreased, whereas D_2cm_ and GI increased.

Figure 3(d) presented the results that when the leaves on both sides of MLC were closed simultaneously (Type 3a) by 1, 2, 3, 4, 5 mm. Compared to the baseline AP plan, the relative percentage changes of PTV were −4.76, −10.07, −15.84, −21.83, −28.11%, respectively. As the Type 3a error was introduced, the value of CI had the largest change from the baseline plan, and the closure error of 1mm could cause a discrepancy of 18.62%. The doses of PTV and OARs decreased by the introduction of the Type 3a error, so did the CI, the nCI and D_2cm_, but GI increased with the error.

The dosimetric effects of the introduction of the system open errors (Type 3b) were opposite to those of the system close errors. As the MLC leaves shifted toward the “open”, CI and D_2cm_ increased, while nCI and GI decreased. The D_mean_ of PTV as well as the D_max_ of spinal cord, the V_10_, V_20_, MLD of total lung was increased by the introduction of the system open errors.

### 3.B Dose sensitivity study of MU‐weighted/unweighted MLC position error of APs

We compared the dose sensitivity of MU‐weighted and unweighted MLC position errors on APs. Table 1 listed the dose sensitivity values of gEUD for PTV, spinal cord, and total lung. Since ideally we would want to take into account the relative weighting for each MLC gap, calculating dose sensitivity based on MU‐weighted regression would be more close to clinical radiotherapy. The result showed that, for all error types of PTV, there were statistically differences in dose sensitivity between the linear fit considering weighted MU or not (*P* < 0.05), indicating MU‐weighted linear regression is significant and necessary. In addition, the correlation coefficient R^2^ of PTV was more than 0.98 in all cases except for the random error scenarios, confirming the authenticity of linear correlation.

**Table 1:** A list of values obtained by linear regression of the gEUD values as a function of MU‐weighted/unweighted MLC error magnitude including the dose sensitivity in units of Gy/mm, R^2^ value, and the *P* value.

Structure	Error type	MU‐weighted		Unweighted		*P* value
		Dose sensitivity	R^2^	Dose sensitivity	R^2^	
gEUD(PTV)	Type1	−4.93	0.48	−0.45	0.48	0.032
	Type2a	−38.94	0.99	−3.42	0.99	<0.001
	Type2b	−41.70	1.00	−3.56	1.00	<0.001
	Type3a	−55.55	1.00	−4.84	0.98	<0.001
	Type3b	30.33	0.99	2.64	0.99	<0.001
gEUD(SC)	Type1	−0.01	−0.33	0.00096	0.04	0.922
	Type2a	4.17	0.96	0.33	0.96	0.189
	Type2b	−0.45	0.86	−0.03	0.81	0.436
	Type3a	−5.73	1.00	−0.49	1.00	0.009
	Type3b	9.62	1.00	0.81	1.00	<0.001
gEUD(TL)	Type1	−0.06	0.21	−0.0055	−0.05	0.150
	Type2a	−0.20	0.99	−0.016	0.98	0.189
	Type2b	0.17	0.48	0.014	0.94	0.228
	Type3a	−2.77	0.99	−0.24	1.00	<0.001
	Type3b	2.88	1.00	0.25	1.00	<0.001

Among three types of MLC error, the dose sensitivity of Type 3b (system close) was the biggest. The gEUD sensitivity values of PTV for system open and close were 30.33 and −55.55 Gy/mm, respectively. Type 2 (system shift) error impacted gEUD slightly, the gEUD sensitivity values of PTV for the left and right shift were −38.94 and −41.70 Gy/mm, respectively. Type 1 (random) errors changed the gEUD negligibly with a dose sensitivity of −4.93 Gy/mm. It is consistent with the DVH shown in Fig. 2 and the pattern of dosimetric parameters shown in Fig. 3. Compared to other misalignments of MLC, the linear relationship between the Type 1 error and gEUD was not strong, the R^2^ value was only 0.48, while other two kinds of errors were greater than 0.98.

The highest dose sensitivity of spinal cord and total lung both appeared for Type 3b error, which was 9.62 and 2.88 Gy/mm, respectively. The dose sensitivity of OARs was relatively small compared to the PTV.

### 3.C Preliminary study of the linear relationship between PTV volume and Type 3 dose sensitivity on APs

We conducted a preliminary investigation of the linear relationship between PTV volume and dose sensitivity of MU‐weighted Type 3 MLC error on APs.

The PTV volume of the different patients and the corresponding dose sensitivity of the PTVs were listed in Table 2. It is evident that as the absolute value of the dose sensitivity tended to decrease substantially with the increase of PTV volume. For example, as the volume of PTV increased from 16.49 cc (case 1 in Table 2) to 159.56 cc (case 10), the dose sensitivity of Type 3a has dropped from −77.30 to −33.87 Gy/mm.

**Table 2:** A list shows the volume of PTV, the corresponding dose sensitivity, and the total MU/per patient for ten patients.

Case	V_PTV_(cc)	Dose sensitivity (Gy/mm)		Total MU/per patient
		Type 3a	Type 3b	
1	16.49	−77.30	44.36	1870
2	17.55	−70.56	39.69	2071
3	25.16	−66.28	37.01	2099
4	47.46	−39.88	27.00	2544
5	49.24	−64.58	27.36	1864
6	53.30	−65.51	24.44	1700
7	55.97	−43.01	26.66	2093
8	62.13	−42.97	26.34	1828
9	90.09	−51.57	28.36	1664
10	159.56	−33.87	22.06	2106

**Fig. 4:**
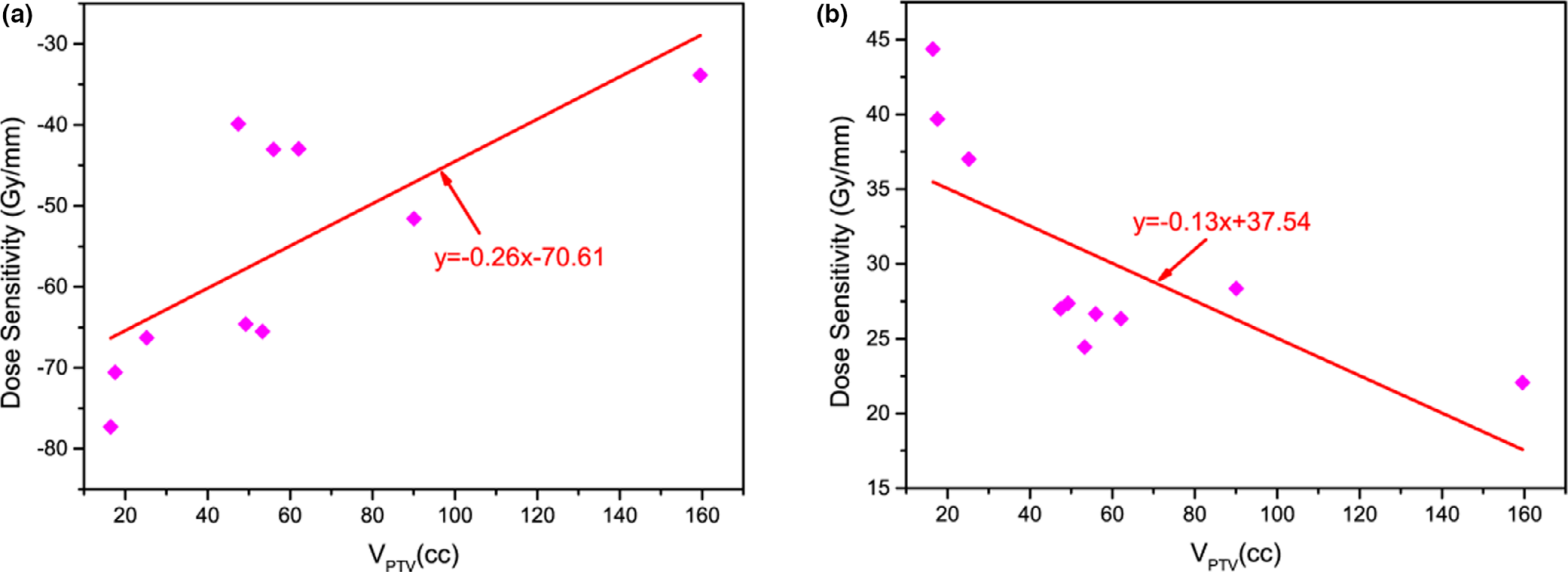
Linear fitting of PTV volume with corresponding Type 3 dose sensitivity.4a: system close, 4b: system open.

Figure 4 showed the linear fitting results of dose sensitivity of Type 3 and the PTV volume. There was a clear linear relationship between them. For Type 3a errors, the linear fitting had a slope of 0.26 and the R^2^ value was 0.48. For Type 3b errors, the slope was 0.13 and the R^2^ value was 0.46. With the increase of PTV volume, the mean MLC gap increased. The influence of open and close errors of MLC on target dose gradually decreased with the increase of PTV volume.

### 3.D Comparison of dose sensitivity between AP and MP

The results of this study showed that the random error had negligible dosimetric effects on the PTV and OARs. Therefore, the comparisons between the AP and MP were focused on Type 2 and Type 3 MLC position errors.

Table 3 listed the dose sensitivity comparisons between AP and MP. For PTV, there were differences in absolute value of dose sensitivity, yet no statistical differences (*P* > 0.05) were found. Compared to the AP, the PTV of MP had larger dose sensitivity to Type 2 and Type 3 MLC position errors. For spinal cord and total lung, the dose sensitivity differences between the MP and the AP were found to be irregular. It is not always the fact that AP has a larger dose sensitivity of OARs than MP.

**Table 3:** Linear regression of planning parameter obtained for Type 2 and Type 3 MLC errors for AP and MP. Parameters listed include the dose sensitivity in units of Gy/mm, the R^2^ value of linear regression, and *P* value.

Structure	Error type	AP		MP		Diff	*P* value
		Dose sensitivity	R^2^	Dose sensitivity	R^2^		
gEUD(PTV)	Type2a	−38.94	0.99	−45.34	0.99	−6.4	0.139
	Type2b	−41.70	1.00	−43.61	0.99	−1.91	0.721
	Type3a	−55.55	1.00	−59.21	1.00	−3.66	0.276
	Type3b	30.33	0.99	35.07	0.98	−4.74	0.290
gEUD(SC)	Type2a	4.17	0.96	3.19	0.87	0.98	0.311
	Type2b	−0.45	0.86	−0.67	0.99	−0.22	0.184
	Type3a	−5.73	1.00	−6.00	1.00	−0.27	0.008
	Type3b	9.62	1.00	8.57	0.98	1.05	0.001
gEUD(TL)	Type2a	−0.20	0.98	−0.24	0.99	−0.04	0.314
	Type2b	0.17	0.95	0.19	0.97	−0.02	0.138
	Type3a	−2.77	1.00	−2.76	1.00	0.01	0.001
	Type3b	2.88	1.00	2.77	1.00	0.11	0.001

*Note:* The column of “Diff” represents for the difference between absolute value of dose sensitivity of APs and MPs.

The example dose calc: Taking the Type 3a MLC position error as an example, the MU‐weighted dose sensitivity is −55.55 Gy/mm, If the MLC position error is 5 mm, Assuming that the total MU is 500 and the MU of a single beam is 50, the corresponding dose deviation is −55.55 Gy/mm × (50 MU/500 MU) × 5 mm = 27.775 Gy.

## 4 DISCUSSION

In clinical IMRT treatment, the accuracy of MLC position is the critical factor to determine the quality of treatment delivery. Especially in fractionally high‐dose radiotherapy of SBRT, the positional error of MLC can cause certain global or local changes in the planned dose distribution. Therefore, the advantages of IMRT cannot be reached without the accurate implementation of MLC position. Oliver et al.^13^ reported that there was a linear relationship between different types of MLC errors and PTV gEUD in VMAT plans of prostate cancer. Sen et al.^21^ reported that when the magnitude of MLC random error in nasopharyngeal carcinoma reaches 2 mm, it has few effects on target and OARs. Blake et al.^22^ reported that there was less patient‐to‐patient variation occurred from MLC delivery uncertainties in VMAT than step‐and‐shoot IMRT. The abovementioned studies on MLC position error were all based on the manual planning. In the current study, we extended the scope and investigated the influence of MU‐weighted MLC position error on dose distribution of SBRT in APs.

This study included the investigation in the influence of MU on dose sensitivity on baseline plans. And for the first time, MU was used in the linear regression fitting for the sensitivity study, confirming the fact that the dose sensitivity of MU‐weighted and unweighted was different. The dosimetric effects of the MLC position errors on the target and OARs were also investigated, as well as the relationship between target volume and magnitude of MLC Type 3 error. It was found that for system open/close MLC errors, as the PTV volume became larger, the dose sensitivity decreased. In addition, we also carried out the work comparing the dose sensitivity of MLC position errors between the AP and the MP, and obtained the preliminary findings that the AP had slightly smaller dose sensitivity of PTV than the MP. Five kinds of MLC position errors were investigated, and it is important to reinforce the necessity of these types of maintenance and tolerance selection. Our research can give inspirations to the commission and maintenance of the linac accelerator.

The relative percentage change between the simulated plan and the baseline AP plan were shown in Fig. 3. Random error (Type 1) had little effect on the target and OARs. The system shift error (Type 2) of MLC was relatively insignificant due to the angle compensation of the beams. The contraction of the MLC gap (Type 3a) could change segment areas of each beam, which could cause changes on the delivery doses. During the entire treatment, the tumor may regress or swell, and the resulting physiological changes are not covered by our study.

Figure 3 shows the relatively sensitive dosimetric parameters (nCI, D2 cm, and CI) for different MLC errors. Since the variations of dose parameters are related to the relative locations of the target and OARs, and the locations vary from patient to patient, the impacts of MLC position errors can be relatively irregular. In clinical situations, some targets are adjacent to the spinal cord or total lung, the slight misalignments of MLC may lead to marked dose changes to the OARs, causing unrecoverable harm to patients. Thus, it is very important to implement adequate quality assurance procedures to assess MLC position errors.

The dosimetric effects of the Type 2a and the Type 2b MLC position errors were inconsistent [Figs. 3(b), 3(c)], and the dose sensitivity of the target and OARs was also not identical, neither was the dose sensitivity of spinal cord and total lung(listed in Table 1). The dose sensitivity of the PTV to the left shift was −38.94 Gy/mm, while to the right shift was −41.70 Gy/mm. It indicated that the dosimetric changes caused by the left and right shifts of MLC banks were not symmetrical. This is because of the asymmetrical shape of the target and asymmetrical relative locations of OARs to the target, leading to asymmetrical shape of the MLC subfields, as well as the asymmetrical dose distributions.

The results of the linear fits of MU‐weighted versus unweighted are listed in Table 1, and statistical differences in dose sensitivity were observed. Except for Type 1, the fitting coefficients R^2^ of PTV were >0.98, confirming the reliability of linear relationship between gEUD of PTV and MLC errors. For random errors, the R^2^ was quite small, and the existence of any difference of dose sensitivity between MU‐weighted and unweighted Type 1 error remains to be further studied.

Even though MU‐weighted MLC position errors presented significant differences on dose sensitivity of PTV, it was not always the same case for OARs. It can be seen from Table 1 that the dose sensitivity of spinal cord and total lung showed less differences between the two kinds of linear fits. In other words, the MU‐weighted MLC position errors seemed to have less impact on OARs than PTV. It is likely that the MU‐weighted MLC position errors are very important in assessing the dose impact of the MLC error on the target, while it is relatively less important to OARs.

Furthermore, the correlation between the dose sensitivity of Type 3 MLC and the PTV volume were evaluated (Table 2). It was found that compared to a small target, a larger target will have a lower dose sensitivity to Type 3 errors. Therefore, in clinical treatment, special attention needs to be paid to ensure accuracy of MLC positions especially for patients with small targets.

Comparing the MP with the AP, we found that the dose sensitivity of PTV of Type 2 and Type 3 MLC in the MP was greater than that of AP. However, since the automatic plan involves multiple dose limiting rings and more complicated calculation algorithms, the difference between the AP and the MP is still unknown. Further study is needed to find the underlying causes.

This study raised a novel mathematical solution to evaluate the MLC position error on dosimetry. The brand‐new MU‐weighted dose sensitivity can provide an approximate dose deviation assessment without the recalculation in TPS. In addition, the index can also be used in log files to calculate the dose deviations of MLC position error for patient treatment. The comparison between different kinds of MLC position errors can provide data for commission and maintenance of linac accelerator. In our study, we found that it is not appropriate to study the MLC deviation without MU, which cannot correctly reflect the dose deviation. The limitation of this paper is that when MLC position error is introduced into the simulation calculation to evaluate the dosimetry difference, the patient motion and setup error are not considered, which is inevitable in clinical practice.

## 5 CONCLUSION

This study investigated the effects of MU‐weighted MLC positional error on dose distribution of SBRT radiotherapy for peripheral NSCLC patients. There is significant difference in dose sensitivity between MU‐weighted and unweighted MLC position errors on APs. Therefore, it is necessary to include MU in the dosimetric evaluation.

## AUTHOR CONTRIBUTIONS

AiHui Feng was involved in conceptualization, investigation, methodology, software, data curation, and writing original draft preparation and review/editing; Hua Chen was involved in conceptualization, methodology, and software; Hao Wang was involved in software and writing review and editing; HengLe Gu was involved in data curation and writing review and editing; Yan Shao was involved in data curation and visualization; YanHua Duan was involved in methodology and visualization; YanChen Ying was involved in software; Ning Jeff Yue was involved in writing review and editing; ZhiYong Xu was involved in conceptualization, methodology, project administration, supervision, and writing original draft preparation and review/editing. All authors read and approved the final manuscript.

## CONFLICT OF INTEREST

None.
